# Genome Sequence of Foot-and-Mouth Disease Virus Serotype O Isolated from Morocco in 2015

**DOI:** 10.1128/genomeA.01746-15

**Published:** 2016-04-21

**Authors:** K. Bachanek-Bankowska, J. Wadsworth, A. Gray, N. Abouchoaib, D. P. King, N. J. Knowles

**Affiliations:** a The Pirbright Institute, Pirbright, Woking, Surrey, United Kingdom; b Laboratoire Regional d’Analyses et de Recherches de Casablanca, ONSSA, Casablanca, Morocco

## Abstract

The genome of a virus isolated from an outbreak of foot-and-mouth disease (FMD) in Morocco in 2015 is described here. This virus is classified as lineage Ind-2001d within serotype O, topotype ME-SA (Middle East-South Asia). This lineage is endemic on the Indian subcontinent but has caused outbreaks in the Middle East and North Africa since 2013.

## GENOME ANNOUNCEMENT

Foot-and-mouth disease (FMD) virus is a single-stranded positive-sense RNA virus of the genus *Aphthovirus* (family *Picornaviridae*), an etiological agent for an economically important vesicular disease that spreads rapidly among susceptible cloven-hoofed animals. Outbreaks of FMD can lead to major economic losses due to reduction in livestock production and disruption in national and international trade. The virus is classified into seven immunologically distinct serotypes, each containing numerous variants that are often restricted to specific geographical locations (topotypes) and lineages (1); due to a high mutation rate, however, new FMD viruses can emerge and challenge local and international disease control strategies.

During November 2015, the FAO World Reference Laboratory for FMD (WRLFMD) received samples from cattle displaying clinical signs of FMD in the Sidi Bennour Province, Morocco. Subsequently, FMD virus (FMDV) serotype O was confirmed to be the causative agent. This was the first detection of FMDV in Morocco since 1999 (2). Phylogenetic analysis revealed relationships of the Moroccan isolates to other viruses in the Middle East-South Asia (ME-SA) topotype, Ind-2001d lineage (O/ME-SA/Ind-2001d) (3). Viruses belonging to this FMDV lineage were originally isolated in the Indian subcontinent, but their emergence in the Middle East (United Arab Emirates and Saudi Arabia) and North Africa (Libya) was reported in 2013 (4, 5), with further spread to Algeria and Tunisia in 2014 (6, 7).

Here, we report the complete genome sequence of an isolate (O/MOR/1/2015) from the O/ME-SA/Ind-2001d lineage causing outbreaks in Morocco in October 2015. This virus was isolated from a clinical sample by a single passage in primary bovine thyroid (BTy) cells. The genome was sequenced using MiSeq technology (Illumina, USA), as previously described (8). Sequence analyses were undertaken using SeqMan NGen and SeqMan Pro version 12 (Lasergene package; DNAStar, Inc., Madison, WI).

The genome sequence of O/MOR/1/2015 was 8,115 nucleotides (nt) in length [including 15 nt of a poly(C) tract of unknown length located within the 5′ untranslated region (UTR)]. The first 6 nt of the genome were not determined. A single large open reading frame of 6,999 nt was predicted to encode a polyprotein of 2,333 amino acids (aa) containing four structural (VP4, VP2, VP3, and VP1) and 10 nonstructural proteins (L, 2A, 2B, 2C, 3A, 3B1, 3B2, 3B3, 3C, and 3D). A comparison to the genome sequence of O/LIB/2/2013 (Libya) belonging to the same lineage (7) showed 96.7% nt identity; however, a deletion of 72 nt was identified in the 5′ UTR. This deletion was not previously reported in other isolates within the Ind-2001d lineage, but deletions in this region of the 5′ UTR have been reported for other FMD viruses (9).

After the initial emergence of the Ind-2001d lineage from the Indian subcontinent to the Middle East and North Africa in 2013, the virus became established in North Africa. The emergence and continued spread of the Ind-2001d lineage reinforce the need to monitor and report outbreaks of FMD not only to understand the global epidemiology of the disease but also to develop tailored control strategies.

### Nucleotide sequence accession number.

The nucleotide sequence of FMDV O/MOR/1/2015 has been deposited in GenBank under the accession no. KU291242.
